# A Pilot Study Evaluating the Contribution of *SLC19A1* (*RFC-1*) 80G>A Polymorphism to Alzheimer's Disease in Italian Caucasians

**DOI:** 10.1155/2014/608104

**Published:** 2014-06-05

**Authors:** Fabio Coppedè, Pierpaola Tannorella, Gloria Tognoni, Silvia Bagnoli, Paolo Bongioanni, Benedetta Nacmias, Gabriele Siciliano, Sandro Sorbi, Ubaldo Bonuccelli, Lucia Migliore

**Affiliations:** ^1^Division of Medical Genetics, Department of Translational Research and New Technologies in Medicine and Surgery, University of Pisa, Medical School, Via Roma 56, 56126 Pisa, Italy; ^2^Unit of Neurology, Department of Neuroscience, Pisa University Hospital, Via Roma 67, 56126 Pisa, Italy; ^3^Department of Neuroscience, Psychology, Drug Research and Child Health (NEUROFARBA), University of Florence, Viale Pieraccini 6, 50139 Florence, Italy; ^4^Unit of Neurorehabilitation, Department of Neuroscience, Pisa University Hospital, Via Paradisa 2, 56124 Pisa, Italy; ^5^Department of Clinical and Experimental Medicine, University of Pisa, Neurological Clinic, Via Roma 67, 56126 Pisa, Italy

## Abstract

Alzheimer's disease (AD) is the most common neurodegenerative disorder and the primary form of dementia in the elderly. Polymorphisms of genes involved in folate metabolism have been frequently suggested as risk factors for sporadic AD. A common c.80G>A polymorphism (rs1051266) in the gene coding for the reduced folate carrier (*SLC19A1* gene, commonly known as *RFC-1* gene) was investigated as AD risk factor in Asian populations, yielding conflicting results. We screened a Caucasian population of Italian origin composed of 192 sporadic AD patients and 186 healthy matched controls, for the presence of the *RFC-1* c.80G>A polymorphism, and searched for correlation with circulating levels of folate, homocysteine, and vitamin B12. No difference in the distribution of allele and genotype frequencies was observed between AD patients and controls. No correlation was observed among the genotypes generated by the *RFC-1* c.80G>A polymorphism and circulating levels of folate, homocysteine, and vitamin B12 either in the whole cohort of subjects or after stratification into clinical subtypes. Present results do not support a role for the *RFC-1* c.80G>A polymorphism as independent risk factor for sporadic AD in Italian Caucasians.

## 1. Introduction

Alzheimer's disease (AD) is the most common neurodegenerative disorder and the primary form of dementia in the elderly, clinically characterized by a progressive neurodegeneration in selected brain regions, including the temporal and parietal lobes and restricted regions within the frontal cortex and the cingulate gyrus [1]. The term “dementia” describes a set of symptoms, which include loss of memory, mood changes, and problems with communication and reasoning. Indeed, AD leads to memory loss accompanied by changes of behaviour and personality severe enough to affect daily life. The disease symptoms get worse over time and available treatments may only help in keeping patients from getting worse for a limited period. It is estimated that there are over 36 million people living with dementia in the world, and projections estimate that the number of affected individuals will increase quickly in the next decades following the worldwide increase in life expectancy. Therefore, there is particular interest in searching for early detectable biomarkers allowing us to better characterize those individuals at increased risk to develop AD [1].

Homocysteine (hcy), folates, and related B-vitamins participate in one-carbon metabolism, a pathway required for DNA synthesis and methylation reactions [2]. Both prospective and retrospective studies suggest that impairments of one-carbon metabolism leading to increased hcy levels might contribute to Alzheimer's disease (AD), and genetic polymorphisms of metabolic enzymes have been suspected to contribute to those impairments as well as to sporadic AD risk [2–16].

The reduced folate carrier (RFC-1) participates in the uptake of folate cofactors from the blood [17], and a common c.80G>A polymorphism (rs1051266) in the gene coding for RFC-1 (*SLC19A1* gene: solute carrier family 19 member 1, commonly known as* RFC-1* gene) was hypothesized to have a functional role in folate transport [18]. Subsequent studies gave conflicting results, and the contribution of this polymorphism to circulating folate or hcy levels is still a matter of debate [15, 19–21]. In 2009, Bi and coworkers observed association of both the* RFC-1* 80G allele and the GG genotype with increased risk of late-onset AD in Han Chinese individuals [15]. However, no significant effect of the* RFC-1* 80G>A polymorphism on plasma folate and hcy levels was detected [15]. A more recent study performed in Indian subjects failed to find association of the* RFC-1* 80G>A polymorphism with risk of AD or vascular dementia, and no association of the polymorphism with serum folate levels was detected [21]. Moreover, others failed to observe association of rs1051266 with cognitive status, folate, and hcy levels in Caucasian Parkinson's disease (PD) patients [22].

At best of our knowledge, except for the above two conflicting studies in Asian populations [15, 21], there is no other available case-control genetic association study evaluating the possible contribution of rs1051266 to AD risk. Therefore, we performed the present pilot study to address the contribution of the* RFC-1* 80G>A polymorphism to AD risk in a cohort of Caucasian sporadic AD patients and healthy matched controls and searched for correlation between rs1051266 and circulating levels of folate, hcy, and vitamin B12.

## 2. Materials and Methods

### 2.1. Study Population

DNA samples from 192 sporadic AD patients and 186 matched controls were collected at the Department of Neurosciences, University of Pisa, and at the Department of Neuroscience, Psychology, Drug Research and Child Health, University of Florence (Table 1). The AD patients were clinically evaluated according to the published guidelines and the AD diagnosis fulfilled the Diagnostic and Statistical Manual of Mental Disorders criteria (DSM-IV) [23, 24]. According to disease age at onset and absence of a family history of dementia all the AD subjects were assumed to be sporadic late-onset (>65 years) cases. The apolipoprotein E (*APOE*) genotype was known for 30 AD patients and 40 controls, and* APOE *ε*4* (+) carriers were higher in AD patients than in controls (47% versus 27%). As normal controls we recruited healthy volunteer subjects without relationship with the AD patients. Controls were selected among people ageing more than 65 years (i.e., people at risk to develop late onset AD) and were matched to AD patients for age (±3 years) and gender (Table 1), as well as for ethnicity and geographic origin (all individuals were Caucasians from northern Tuscany and neighbouring areas). Family history of dementia was ascertained, excluding all the subjects with even one relative who developed AD or other dementias. All the control subjects were evaluated in order to exclude the presence of cognitive impairment (MMSE score over 26). Each subject gave an informed and written consent for genotype analysis. The study was performed in accordance with the Declaration of Helsinki and approved by the Ethics Committee of the Pisa University Hospital (Protocol number 3618/2012).

### 2.2. Genotyping

Genomic DNA was isolated from whole blood by means of the QIAamp Blood Mini Kit (Qiagen, Milan, Italy) following the manufacturer's instructions. The genotyping protocol for the* RFC-1* 80G>A polymorphism was adapted from Bi et al. [15]: a 230-bp product was amplified using 1.25 units of Taq DNA polymerase (Invitrogen, Milan, Italy), 10 pmol of the forward primer (5′-AGCGTCACCTTCGTCCC-3′) and the reverse primer (5′-TCCCGCGTGAAGTTCTTG-3′), 0.15 mM of each dNTP, 1.5 mM MgCl_2_, and 30 ng of genomic DNA in a total volume of 25 *μ*L. PCR conditions consisted of an initial denaturation step of 5 minutes at 95°C, followed by 40 cycles of 30 s at 95°C, 45 s at 62°C, and 45 s at 72°C and a final extension of 10 minutes at 72°C. The PCR products were digested with* Cfo I* (SIGMA, Milan, Italy) and resulted in three fragments of 125-bp, 68-bp, and 37-bp in the presence of the 80G allele, while the 80A allele produced two fragments of 162- and 68-bp. Digestion products were visualized after electrophoresis on a 3% agarose gel containing ethidium bromide. Internal control samples, whose genotypes had been previously assessed, were always included and analyzed on each gel.

### 2.3. Biochemical Analyses

Peripheral blood samples for the evaluation of folate, total homocysteine (t-hcy), and vitamin B12 levels were collected from 104 AD patients and 64 healthy controls. Plasma was immediately separated and stored in freezer at −80°C. All the analyses were performed with standard protocols at the diagnostic laboratory of the University Hospital of Pisa, as previously described by us [5]. In order to minimize the effect of polymedication in our cohort of subjects, individuals taking medicaments or supplements known or suspected to interfere with one-carbon metabolism were not enrolled for the study.

### 2.4. Statistical Analyses

To verify that allele frequencies were in Hardy-Weinberg equilibrium and to assess differences in allele distributions between groups, we used the Chi-square (*χ* ^2^) analysis. The differences in genotype frequencies were analyzed by 2 × 2 contingency tables using *χ* ^2^ analysis. Logistic regression analyses were used to examine the associations between the study polymorphism and AD risk by estimating odds ratios (ORs) and 95% confidence intervals (CIs) with and without adjustment for age and gender. All individual values were analyzed with the MedCalc 12.5 statistical package for Windows. The statistical package QUANTO 1.2.4.exe was used to evaluate the statistical power of the study. Given a case-control cohort of almost 190 subjects each and a minor allele frequency ranging from 0.45 to 0.49 from present and previous studies in Caucasians [25, 26], the study had 80% power to detect ORs of 1.5 or higher under the additive model.

Analysis of covariance (ANCOVA) was used to evaluate differences in mean plasma t-hcy, folate, and vitamin B12 levels among groups, including age at sampling and gender as covariates. ANCOVA was also used to correlate biochemical data with the possible genotypes generated by the studied polymorphism, including age and gender as covariates. Since data on t-hcy, folates, and vitamin B12 had a skewed distribution, logarithm transformation of all values was done before analysis. Analyses were performed with the Statgraphics Centurion XVI.1 software package for Windows.

## 3. Results

### 3.1. *RFC-1* Allele and Genotype Frequencies among Groups

Table 2 shows the distribution of* RFC-1* 80G>A genotype and allele frequencies in AD patients and controls. Genotype distributions in controls conformed to Hardy-Weinberg expectations (*P* = 0.44). The frequencies of the* RFC-1* 80A minor allele in AD patients and controls were 0.46 and 0.47, respectively (*P* = 0.69). Also the distribution of genotype frequencies was similar and not significantly different between AD and control subjects (Table 2). As stated in the Materials and Methods section the* APOE* genotype was known only for a small subgroup of AD and control individuals. However, no significant difference in* RFC-1* 80G>A allele frequencies was observed between* APOE* *ε*4 (+) AD patients and* APOE* *ε*4 (+) controls (*P* = 0.37. Not shown).

### 3.2. Folate, t-hcy, and Vitamin B12 Levels among Groups

Data on circulating t-hcy, folate, and vitamin B12 levels were available from 104 AD and 64 control individuals (Table 1). Analysis of variance revealed that mean t-hcy levels were higher in AD patients than in controls (*P* = 0.002), but after inclusion of age at sampling and gender as covariates in the analysis the difference between AD and control subjects was not statistically significant (*P* = 0.14), whilst a strong effect of age at sampling on increasing t-hcy levels was observed (*P* < 0.001). A significant difference was observed concerning serum folate levels between AD and control subjects (*P* = 0.01) that remained significant after correcting for age at sampling and gender (*P* = 0.04). Also increasing age at sampling showed a significant contribution to reducing serum folate levels in our population (*P* = 0.03). No difference in mean vitamin B12 levels was observed between AD and controls (*P* = 0.31 without correction and *P* = 0.47 after correction for age and gender). No significant effect of age and gender on mean vitamin B12 levels was observed.

### 3.3. Correlation between* RFC-1* Genotypes and Biochemical Data

Table 3 shows the correlation between* RFC-1* 80 (GG, GA, AA, and GA+AA) genotypes and circulating levels of t-hcy, folate, and vitamin B12. Analyses were performed in the whole cohort of subjects (AD + controls) and in AD and control individuals separately. No significant difference was observed for each of the studied biochemical markers among different* RFC-1* genotypes (Table 3).

## 4. Discussion

At best of our knowledge the present is the first case-control study performed in Caucasians and aimed at addressing the contribution of the* RFC-1* 80G>A polymorphism to late-onset AD risk. The study revealed no significant difference in* RFC-1* allele or genotype frequencies between late-onset AD patients and healthy matched controls, both results being very similar between the two groups (Table 2). In addition, no significant effect of the studied polymorphism on circulating levels of folate, vitamin B12, or t-hcy was observed (Table 3).

In their original report, Bi and coworkers included 275 late-onset AD patients and 271 age-matched controls observing an additive effect for the G allele and odds ratios (ORs) ranging from 1.4 to 1.6 for genotype comparisons. The present study had enough power to detect similar ORs under an additive genetic model or at least to detect some trends toward an association. However, both allele and genotype frequencies were closely similar between AD patients and controls; the ORs for genotype comparison were close to 1.0 and the respective *P* values did not even suggest trends for association. Therefore, rs1051266 is unlikely to represent an independent risk factor for sporadic AD in our population, at least with a similar effect size as previously reported in Han Chinese individuals [15]. Furthermore, present results are in agreement with those of Mansoori and coworkers who screened 80 AD patients, 50 patients with vascular dementia, and 120 healthy control subjects from India, observing an increased risk of dementia in subjects with low serum folate values but no association of rs1051266 with circulating folate levels and risk of AD or vascular dementia [21]. In addition, Białecka and coworkers [22] screened 248 PD individuals and 254 matched controls from Poland, searching for correlation between rs1051266 and risk of dementia in Parkinson's disease. The authors observed that both age and plasma hcy levels were risk factors for dementia in PD but failed to find association of the *RFC-1* 80G>A polymorphism with cognitive decline or plasma hcy levels [22]. Similarly, Kumudini and coworkers [27] recently screened a cohort of 151 Indian PD patients and 416 healthy controls, observing increased plasma hcy levels in PD patients than in controls but no association of the *RFC-1* 80G>A polymorphism with either PD risk or plasma hcy levels [27].

Taken overall, both the present and the four previous studies performed in individuals with different forms of dementia or neurodegeneration [15, 21, 22, 27] failed to find association of the *RFC-1* 80G>A polymorphism with circulating folate, hcy, or vitamin B12 levels, and only one study [15] suggests association with dementia of Alzheimer's type.

Several factors could account for the above conflicting results, including differences in allele frequency, dietary habits, environmental and geographic factors, and the presence or absence of other genetic variants. For example, the frequency of the alleles generated by the* RFC-1* 80G>A polymorphism varies among different populations, with the* RFC-1* G allele often reported to be the major allele in certain populations [18, 21, 25] and the minor allele in others [15, 28]. Dietary regimens rich in folate, such as the Mediterranean diet, could mask the effect of certain polymorphisms, as it happens for the* MTHFR* 677C>T one, the most studied polymorphism of the folate pathway, which is associated with increased risk of sporadic AD in Asians but not in Caucasians [16]. It was also suggested that geographic factors, such as the latitude, could interfere with ultraviolet B solar radiation and promote, in less pigmented skins, intravascular folate photolysis, thereby affecting circulating folate levels and folate metabolism [29]. In this regard, a recent literature meta-analysis reported a significant effect of the* MTHFR* 677C>T polymorphism on pregnancy outcome only in subtropical regions [29], and it is also of interest that the* RFC-1 *80G>A polymorphism was associated with increased chromosome damage in blood cells of healthy Australian individuals but not in those of healthy Italian ones [30, 31]. Moreover, the presence/absence of other polymorphisms of the pathway could mask or potentiate the effect of a single one [26]. Altogether those factors can account for a different weight of each genetic polymorphism on a given disease among different populations, and also the* APOE* *ɛ*4 variant, which is the most known and replicated risk factor for sporadic AD, seems to confer different relative risks in different ethnic groups [32].

Interestingly, we observed reduced serum folate levels in AD patients with respect to controls, and this is in agreement with several recent reports suggesting that reduced serum folate levels are a valuable biomarker of AD in aged individuals and might be linked to increased atrophy of both cortical and subcortical regions [21, 26, 33, 34]. However, as discussed above, neither the present nor the previous studies observed association of the* RFC-1* 80G>A polymorphism with serum folate levels in individuals affected by different forms of dementia or neurodegeneration [15, 21, 22, 27].

A limitation of the present study is that patients were selected retrospectively among prevalent AD cases followed up at our neurological clinics. The cognitive decline leading to AD usually starts several years before the onset of dementia, a condition which is referred to as mild cognitive impairment (MCI) [35]. The analysis of individuals with MCI would help to better clarify factors linked to the earliest phases of the disease than the analysis of late onset AD cases [35], and a similar study is highly desired in order to clarify the contribution of both present and other polymorphisms of the folate metabolic pathway to the earliest phases of the neurodegenerative process leading to AD. MCI patients should, however, be followed up over a period of time in order to discriminate those that will develop dementia of AD type from other forms of dementia. Indeed, the question of whether or not impairments of the folate metabolic pathway are cause or consequence of the neurodegenerative process in AD is still open in the literature [8–10]. In order to minimize factors, such as polymedications, that could interfere with the measured values of folate, hcy, and vitamin B12, we have not included in the present study patients or controls taking drugs or vitamin supplements known to alter those metabolites. In addition, in order to minimize the effect of geographic factors, both cases and controls had the same geographical origin and were residents of Pisa, Florence, and neighbouring areas at the time of enrolment for the study. Another limitation that we should acknowledge is that we had no opportunity to measure folate, hcy, and vitamin B12 in the whole cohort of subjects but only in a subgroup of them. As a consequence, data shown in Table 3 should be taken with caution, and replication in a larger cohort of Italian elderly subjects is warranted prior to exclude a role for the studied polymorphism on circulating folate, hcy, and vitamin B12 levels. In this regard, a large similar study, performed in over 1.000 elderly English subjects (mean age 77.9 years), revealed no association of the* RFC-1* 80G>A polymorphism with circulating folate or hcy levels [36], supporting present and previous observations in patients with neurodegenerative diseases and their matched controls [15, 21, 22, 27].

In conclusion, the present pilot study does not support a role for the* RFC-1* 80G>A polymorphism as independent risk factor for sporadic AD in Italian Caucasians. Despite that the group of patients and controls was relatively small, both allele and genotype frequencies results were so similar between AD and control samples that not even a trend for association was detected. Furthermore, no functional contribution of the studied polymorphism on circulating levels of folate, t-hcy, and vitamin B12 was observed. However, a large prospective study is warranted to confirm the results of the present pilot study and exclude a role for this polymorphism in the onset of dementia of Alzheimer's type in Italian Caucasians.

## Figures and Tables

**Table 1:** Demographic characteristics of the study population and data on plasma total homocysteine and serum folate and vitamin B12 levels.

	*n*	Males *n* (%)	Females *n* (%)	Age (years, mean ± SD)	t-hcy^a^ (*μ*mol/L, mean ± SEM)	Folate^a^ (ng/mL, mean ± SEM)	Vitamin B12^a^ (pg/mL, mean ± SEM)
AD	192	65 (33.8%)	127 (66.2%)	76.4 ± 7.3	21.2 ± 1.7	7.1 ± 0.8^b^	407.8 ± 25.2
Controls	186	72 (38.7%)	114 (61.3%)	73.5 ± 6.4	14.6 ± 0.7	8.2 ± 1.0	437.9 ± 30.9

^a^Available from 104 AD and 64 controls.

^b^Significant difference versus controls (*P* value obtained with analysis of covariance using log transformed data and corrected for age and gender).

**Table 2:** Distribution of genotypes and allele frequencies of the *RFC-1 *80G>A polymorphism in Alzheimer's disease and control individuals.

Genotypes/alleles	AD patients *n* = 192 (%)	Controls *n* = 186 (%)	Crude OR (95% CI)	*P* value	Adjusted OR^a^ (95% CI)	*P* value
Genotypes						
GG	53 (27.6)	49 (26.3)	1.00^b^	—	1.00^b^	—
GA	102 (53.1)	98 (52.7)	0.96 (0.59–1.55)	0.87	0.97 (0.58–1.62)	0.89
AA	37 (19.3)	39 (21.0)	0.88 (0.48–1.59)	0.66	0.94 (0.50–1.78)	0.86
AA + GA versus GG	139 (72.4)	137 (73.7)	0.94 (0.60–1.48)	0.78	0.96 (0.59–1.57)	0.89
Alleles						
Allele G	0.54	0.53	1.00^b^	—		—
Allele A	0.46	0.47	0.94 (0.71–1.25)	0.69	0.98 (0.72–1.32)	0.88

^a^Adjusted for age and gender.

^b^Reference value for OR.

**Table 3:** Correlation between *RFC-1 *80G>A genotypes and biochemical data.

	*RFC-1* GG	*RFC-1* GA	*RFC-1* AA	*RFC-1* GA + AA	*P* value^a^
Total (*n* = 168)	39	95	34	129	
Folate (ng/mL, mean ± SEM)	8.4 ± 1.2	7.9 ± 0.8	5.8 ± 1.3	7.3 ± 0.7	0.51
t-hcy (*μ*mol/L, mean ± SEM)	15.6 ± 2.2	19.2 ± 1.4	19.1 ± 2.2	19.1 ± 1.2	0.55
Vit. B12 (pg/mL, mean ± SEM)	462.1 ± 39.3	421.7 ± 24.9	366.1 ± 41.6	406.9 ± 21.4	0.63
AD (*n* = 104)	26	60	18	78	
Folate (ng/mL, mean ± SEM)	8.1 ± 1.5	7.4 ± 1.0	4.9 ± 1.8	6.8 ± 0.9	0.65
t-hcy (*μ*mol/L, mean ± SEM)	16.1 ± 3.5	22.6 ± 2.2	22.1 ± 4.1	22.5 ± 2.0	0.50
Vit. B12 (pg/mL, mean ± SEM)	467.4 ± 47.9	398.7 ± 31.6	351.7 ± 57.5	387.7 ± 27.6	0.70
Controls (*n* = 64)	13	35	16	51	
Folate (ng/mL, mean ± SEM)	8.3 ± 2.2	8.5 ± 1.3	7.1 ± 1.9	8.1 ± 1.1	0.75
t-hcy (*μ*mol/L, mean ± SEM)	13.2 ± 1.4	14.3 ± 0.8	16.6 ± 1.2	15.0 ± 0.7	0.17
Vit. B12 (pg/mL, mean ± SEM)	407.9 ± 70.7	457.8 ± 40.8	404.2 ± 60.9	441 ± 33.7	0.76

^a^ *P* value obtained with analysis of covariance using log transformed data and corrected for age and gender.
